# High Rates of All-cause and Gastroenteritis-related Hospitalization Morbidity and Mortality among HIV-exposed Indian Infants

**DOI:** 10.1186/1471-2334-11-193

**Published:** 2011-07-15

**Authors:** Harjot K Singh, Nikhil Gupte, Aarti Kinikar, Renu Bharadwaj, Jayagowri Sastry, Nishi Suryavanshi, Uma Nayak, Srikanth Tripathy, Ramesh Paranjape, Arun Jamkar, Robert C Bollinger, Amita Gupta

**Affiliations:** 1 Division of Infectious Diseases, Johns Hopkins University School of Medicine, 600 N. Wolfe Street, Baltimore, 21205, USA; 2 Johns Hopkins University-Byramjee Jeejeebhoy Medical College Clinical Trial Unit, Jaiprakash Narayan Road, Pune, 411001, India; 3 Department of Pediatrics, Byramjee Jeejeebhoy Medical College, Jaiprakash Narayan Road, Pune, 411001, India; 4 Department of Clinical Research & Development, Byramjee Jeejeebhoy Medical College, Jaiprakash Narayan Road, Pune, 411001, India; 5 University of Virginia, Center for Public Health Genomic, West Campus, Charlottesville, 22908, USA; 6 National Aids Research Institute, 73 G Block, Bhosari, Pune, 411026, India; 7 Maharashtra Universities of Health Sciences, Nasik, 422004, India; 8 Division of Infectious Diseases, Weill Cornell Medical College, New York City, NY USA

**Keywords:** Infant, HIV, India, Hospitalization, In-hospital Mortality, gastroenteritis, pneumonia

## Abstract

**Background:**

HIV-infected and HIV-exposed, uninfected infants experience a high burden of infectious morbidity and mortality. Hospitalization is an important metric for morbidity and is associated with high mortality, yet, little is known about rates and causes of hospitalization among these infants in the first 12 months of life.

**Methods:**

Using data from a prevention of mother-to-child transmission (PMTCT) trial (India SWEN), where HIV-exposed breastfed infants were given extended nevirapine, we measured 12-month infant all-cause and cause-specific hospitalization rates and hospitalization risk factors.

**Results:**

Among 737 HIV-exposed Indian infants, 93 (13%) were HIV-infected, 15 (16%) were on HAART, and 260 (35%) were hospitalized 381 times by 12 months of life. Fifty-six percent of the hospitalizations were attributed to infections; gastroenteritis was most common accounting for 31% of infectious hospitalizations. Gastrointestinal-related hospitalizations steadily increased over time, peaking around 9 months. The 12-month all-cause hospitalization, gastroenteritis-related hospitalization, and in-hospital mortality rates were 906/1000 PY, 229/1000 PY, and 35/1000 PY respectively among HIV-infected infants and 497/1000 PY, 107/1000 PY, and 3/1000 PY respectively among HIV-exposed, uninfected infants. Advanced maternal age, infant HIV infection, gestational age, and male sex were associated with higher all-cause hospitalization risk while shorter duration of breastfeeding and abrupt weaning were associated with gastroenteritis-related hospitalization.

**Conclusions:**

HIV-exposed Indian infants experience high rates of all-cause and infectious hospitalization (particularly gastroenteritis) and in-hospital mortality. HIV-infected infants are nearly 2-fold more likely to experience hospitalization and 10-fold more likely to die compared to HIV-exposed, uninfected infants. The combination of scaling up HIV PMTCT programs and implementing proven health measures against infections could significantly reduce hospitalization morbidity and mortality among HIV-exposed Indian infants.

## Background

Pneumonia and gastroenteritis account for nearly 50% of early childhood morbidity and mortality, particularly in low-income country settings [1]. HIV-infection increases the risks of these infections several fold and of mortality approximately 9-fold in sub-Saharan Africa [2-5]. Some studies have shown that HIV exposure, even in the absence of infant HIV infection, also increases these risks [6,7]. Most of these studies have defined morbidity as infections, severe events, or grade 3 or 4 adverse events using US NIH Division of AIDS toxicity scale; few have used hospitalization as an outcome. Hospitalization is an important measure of morbidity because it provides discrete information about illness severity, may be more comparable across studies than morbidity events, and is associated with high mortality, particularly among hospitalized HIV-infected infants not on HAART [5].

India has the third highest number of HIV infections in the world, with an estimated 21,000 newly HIV-infected infants [8] and 180,000 HIV-exposed, uninfected infants born each year [9,10]. Examining hospitalization morbidity among HIV-infected and HIV-exposed, uninfected Indian infants is necessary to determine country-specific feasible and sustainable targets to further reduce morbidity and mortality. Therefore, we used data from Six Week Extended-Dose Nevirapine (SWEN), a recently completed NIH randomized clinical trial of prevention of mother-to-child HIV transmission (PMTCT) among breastfed infants in Pune, India [11] to investigate all-cause hospitalization rates among HIV-infected and HIV-exposed, uninfected infants. We also examined cause-specific hospitalization rates, as well as hospitalization reasons, and risk factors for hospitalization among HIV-infected and HIV-exposed, uninfected infants. We specifically sought to compare the impact of SWEN on infant hospitalization rates as the PMTCT trial had earlier shown reduced mortality rates among infants who received SWEN compared to single-dose nevirapine (sdNVP) and because SWEN has been incorporated into the new WHO PMTCT guidelines [12]. We hypothesized that respiratory-related hospitalizations would predominate throughout the first 12 months of life and the proportion of gastrointestinal-related hospitalizations would increase over the first 12 months of life.

## Methods

### Study Design

This is a secondary analysis of the infant outcomes collected as part of the SWEN trial, which was conducted from August 2002 to September 2007. Full details of the parent study have been previously described [12]. In brief, all infants received standard of care (sdNVP) at birth and then were randomized to receive either the daily study intervention of nevirapine plus multivitamins from day 8 to 42 of life or multivitamins alone from day 8 to 42. Women with the intention to exclusively breastfeed were enrolled and encouraged to rapidly wean after the first four months of infant life, which was the local standard of care during this study for women who did not have access to safe, affordable, sustainable formula feeding options [13]. Written consent was obtained where possible; for eligible women who could not read, consent was obtained orally and documented in writing by a witness. Institutional Review Boards in Pune, India and Baltimore, United States approved the study.

### Study Population

Pregnant women, >18 years, were recruited from the antenatal clinic, labor ward, or postpartum ward within 72-hours of delivery at Byramjee Jeejeebhoy Medical College (BJMC)/Sassoon Hospital in Pune, India. All randomized singleton and twin infants born to HIV-infected women were included.

### Study Follow-up

Infant study visits were scheduled at 1, 2, 3, 4, 5, 6, 10, and 14 weeks, and 6, 9, and 12 months of life. Heel-stick and venipuncture blood specimens were collected for HIV testing within 48 hours of birth and at each follow-up visit except weeks 3 and 5, until diagnosis was confirmed. At each scheduled and illness visit, medical history, physical exam, and laboratory studies were performed. Infants were offered cotrimoxazole prophylaxis at 4-6 weeks of life for primary prevention of *Pneumocystis jirovecii *pneumonia (PCP). All infants were followed for 12 months. No vaccines were given as part of this study. Infants and their mothers received routine pediatric health care from their own providers, including any recommended vaccinations, which at the time of the study did not include Hib, pneumococcal or rotavirus vaccines. HAART was not available through the government program during this study so only infants who were HAART eligible and whose families could afford to pay for HAART out-of-pocket received HAART.

### Study Definitions and Analysis

The primary outcome of this study was all-cause 12-month hospitalization rate among HIV-exposed infants. Secondary outcomes included cause-specific 12-month hospitalization and in-hospital mortality rates according to HIV status, as well as reasons, and risk factors for hospitalization.

#### Hospitalization and Mortality

Hospitalization was defined as any overnight admission for any reason during the first 12 months of life. Three cause-specific reasons for hospitalization were evaluated: gastroenteritis, sepsis/meningitis, and pneumonia. WHO guidelines for presumed or probable clinical diagnosis were used by staff physicians [14]. All hospitalizations were verified by the study team, including those at hospitals other than the study site hospital. Hospitalization event data were extracted from electronic medical records of adverse events reported as part of the clinical trial. Death was documented by a physician if it occurred at the hospital or verbal autopsy if the death occurred at home. In-hospital mortality was defined as any death that occurred during hospitalization.

#### HIV Status

HIV status was categorized as infected if a positive DNA PCR test was confirmed by an HIV-1 RNA PCR test (>5,000 copies/ml) at a subsequent visit. All test results underwent external quality assurance as described elsewhere [12].

#### Infant Variables

Cotrimoxazole use was categorized based on adherence to WHO guidelines [15]. Duration of any breastfeeding was determined by the last visit date of ongoing breastfeeding and was divided into 3 categories based on WHO guidelines [16].

#### Maternal Variables

The maternal CD4 cell count, viral load, and hemoglobin collected closest to delivery were used. Hemoglobin categories were based on WHO guidelines[17]. Delivery type was categorized as vaginal (spontaneous, breech, or forceps assisted) or surgical (vacuum extraction, elective, or emergent caesarian section). Maternal HAART use included HAART prescribed during any point of the study.

### Statistical Analysis

Baseline characteristics of the infants and their mothers, as well as, reasons for hospitalizations were summarized using descriptive statistics. Means with standard deviations were used for normally distributed data; medians with interquartile range (IQR) were used for non-normal distributions. For normally distributed data, the means were compared using a Student's t-test and for non-normal data, medians were compared with non-parametric tests like Mann-Whitney. Proportions were compared using chi-squared or Fisher's exact test, whichever was appropriate. Ninety-five percent confidence intervals were used and two-sided p < 0.05 was considered significant.

If infants were hospitalized multiple times, each hospitalization was counted separately in the rate calculations. If an infant received multiple diagnoses during one hospitalization, a single reason for hospitalization was recorded according to the following order: meningitis/sepsis > pneumonia > gastroenteritis > tuberculosis > syphilis. Hospitalization recorded as sepsis included those due to pneumonia, gastroenteritis, or other causes. Risk rates were compared using Poisson regression.

Univariate Poisson regression analyses were performed with infant and maternal predictors with all-cause hospitalization. Selection of risk factors for multivariate analysis, using Poisson regression, was based on biological significance and statistical significance in the univariate analysis. Data were analyzed using STATA version 10 [18]. This study is registered with ClinicalTrials.gov, number NCT00061321.

## Results

### Study Population

A total of 737 infants contributed to 695 person-years (PY) of follow-up; 93 (13%) infants were HIV-infected, and 15 (16%) were on HAART by 12 months of life. Although both groups were counseled the same, a higher percentage of HIV-infected infants received cotrimoxazole (66% vs. 38%, p < 0.001) and were breastfed for a longer duration (171 vs. 101 days, p = 0.03) compared to HIV-exposed, uninfected infants (Table 1). Mothers of HIV-infected infants had lower median maternal CD4 cell counts (366 vs. 494, p = 0.01) and higher log viral loads (4.56 vs. 3.73, p < 0.001) closest to the time of delivery, were more likely to be on HAART (15% vs. 8%, p = 0.01) at any time during the study, and had higher gravidity compared to mothers of HIV-exposed, uninfected infants (Table 1).

**Table 1:** Infant and maternal baseline characteristics among 737 HIV-exposed infants by infant HIV status at 12 months of life in Pune, India

	Total N	HIV- infected N = 93	HIV-exposed, uninfected N = 644	P-value^‡^
**Infant Characteristics**				
Female gender, n (%)	737	46 (49)	301 (47)	0.62
Gestational age (weeks), median (IQR)	732	39 (38-40)	39 (38-40)	0.13
APGAR at 5 minutes, median (IQR)	737	8 (8-9)	8 (8-9)	0.48
Birth weight (grams), median (IQR)	732	2550 (2450-2950)	2650 (2400-3000)	0.29
WHO-guided Cotrimoxazole use	737	61 (66)	247 (38)	<0.001
Any breastfeeding (days), median (IQR)	718	171 (98-276)	101 (98-182)	0.03
<4 months, n (%)	398	42 (45)	356 (57)	
4 - 6 months, n (%)	75	6 (6)	69 (11)	0.02
>6 months, n (%)	264	45 (48)	219 (34)	
Extended dose nevirapine, n (%)	737	41 (44)	326 (51)	0.24
Infant HAART, n (%)†	737	15 (16)	n/a	n/a

**Maternal Characteristics**				
Age (years), median (IQR)	733	24 (21-26)	23 (21-25)	0.09
Gravidity, median (IQR)	619	2 (1-3)	2 (1-3)	0.01
Married, n (%)	733	90 (97)	624 (97)	>0.99
Education	737			
Primary (<6 years), n (%)	294	44 (48)	250 (39)	
Secondary (6-10 years), n (%)	376	39 (42)	337 (52)	0.17
Tertiary (>10 years), n (%)	67	10 (12)	57 (9)	
Hindu religion, n (%)	733	76 (82)	493 (77)	0.27
Vaginal delivery, n (%)	737	77 (83)	513 (80)	0.48
Hemoglobin*	737			
Normal (>11 g/dl), n (%)	364	36 (38.7)	328 (50.9)	
Mild Anemia (10-11 g/dl), n (%)	210	12 (12.9)	90 (14.0)	0.06
Moderate Anemia (7-10 g/dl), n (%)	140	35 (37.6)	188 (29.2)	
Severe Anemia (<7 g/dl), n (%)	23	10 (10.8)	38 (5.9)	
CD4 cell count (cells/mm^3^),median (IQR)*	636	366 (217-549)	494 (334-682)	0.01
Viral load (Log 10) (copies/ml), mean (SD)*	682	4.56 (0.9)	3.73 (1.0)	<0.001
Maternal HAART, n (%)†	737	14 (15)	48 (8)	0.01
Maternal AZT, n (%)^≠^	737	224 (35)	24 (26)	0.09
Maternal NVP, single-dose, n (%)^≠^	737	54 (58)	431 (67)	0.09

NOTES:

*Values closest to delivery are reported.

†HAART use during any time of the study.

‡ P-values based on chi-square, Fisher's exact, Median test or un-paired t-test.

^≠^AZT = zidovudine for PMTCT, NVP = nevirapine (intrapartum, single-dose) for PMTCT.

**Figure 1:**
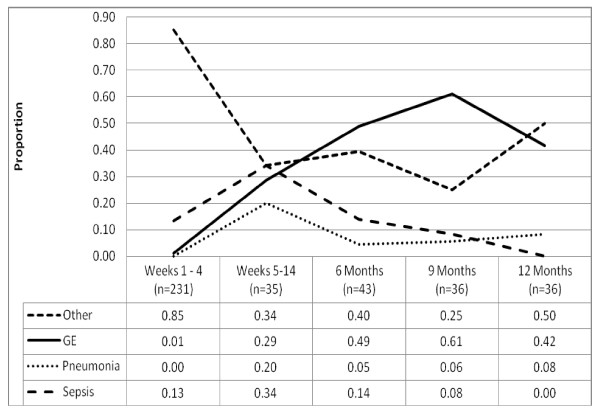
**Other reasons for hospitalization include infections, other than those specifically listed, as well as non-infectious reasons**. The full list of reasons is reported in **Table 3**.

Among the 737 infants, 260 (35%) were hospitalized at least once for a total of 381 hospitalizations. Eighty (30%) of these 260 were hospitalized multiple times (range 2-6). HIV-infected infants were more likely to be hospitalized once (47% vs. 34%, p < 0.05) and multiple times (20% vs. 9%, p < 0.05); however median duration of first hospitalization (6 days vs. 7 days, p = 0.48) and annual days of hospitalization (8 days vs. 6 days, p = 0.79) were similar to that of HIV-uninfected infants. Sepsis/meningitis and pneumonia-related hospitalization frequency increased until 14 weeks of life, then decreased. Gastrointestinal-related hospitalizations steadily increased over time, peaking around 9 months (Figure 1). Other reasons for hospitalization (non-infectious or infections other than gastrointestinal, respiratory, sepsis/meningitis) were highest in the neonatal period and decreased thereafter.

### Hospitalization Rates

The overall all-cause hospitalization rate was 548/1000 PY (95% CI: 495/1000 PY- 606/1000 PY). HIV-infected infants were 1.82-fold more likely to be hospitalized for any reason (906/1000 PY vs. 497/1000 PY, 95% CI: 1.40-2.34) and were more likely to be hospitalized for each of the cause-specific infections, particularly gastroenteritis (229/1000 PY vs. 107/1000 PY, RR 2.14, 95% CI: 1.23-3.58) compared to HIV-exposed, uninfected infants (Table 2). However, there were no statistically significant differences in all-cause or cause-specific hospitalization rates according to infants who received single dose versus six weeks of extended dose of nevirapine prophylaxis (SWEN) (data not shown).

**Table 2:** Hospitalization and mortality rates among 737 HIV-exposed infants at 12 months of life in Pune, India

	Overall		HIV-infected		HIV-exposed, uninfected		Rate Ratio	
	N = 737		N = 93		N = 644			
	
	cases	Rate/1000 PY (95% CI)	cases	Rate/1000 PY (95% CI)	cases	Rate/1000 PY (95% CI)	(95% CI)	P-value
**Hospitalization**								
All-cause	260	548 (495-606)	44	906 (717-1129)	216	497 (443-556)	1.82 (1.40-2.34)	<0.001
Cause-specific								
Gastroenteritis	65	122 (98-151)	16	229 (140-354)	49	107 (83-136)	2.14 (1.23-3.58)	<0.05
Sepsis/Meningitis	44	63 (46-85)	7	103 (47-196)	33	58 (40-80)	1.79 (0.76-3.80)	0.14
Pneumonia	23	35 (22-51)	5	57 (28-63)	18	31 (19-49)	1.83 (0.53-5.07)	0.25
**Mortality**								
In-Hospital^†^	5	7 (2-17)	3	35 (7-103)	2	3 (0.4-12)	10.3 (1.18-123.07)	0.02
Overall	27	40 (27-59)	13	152 (81-261)	14	24 (13-40)	6.36 (2.75-14.60)	<0.001

NOTES:

Case was defined as the number of infants experiencing the event. All-cause hospitalization includes any reason for hospitalization. The causes of death for the 5 infants who died in-hospital include sepsis, pneumonia (2), gastroenteritis, and jaundice.

### In-Hospital Mortality

Overall, 27 (4%) infants died during the 12-month follow-up; 13/93 were HIV-infected infants and 14/644 were HIV-exposed, uninfected infants (14% vs. 2%, p < 0.05). Five (1.9%) of 260 hospitalized infants died in the hospital during the 12-month follow-up; 3/44 hospitalized infants were HIV-infected and 2/216 were HIV-exposed, uninfected (6.8% vs. 0.9%, p = 0.01). The median length of total hospitalization among infants who died in the hospital was greater than those who did not die in the hospital but this did not reach statistical significance (8.5 days vs. 5.5 days, p = 0.46). The causes of death among these 5 infants were gastroenteritis, sepsis, jaundice, and pneumonia. The overall and in-hospital mortality rates were 6.36-fold (152/1000 PY vs. 24/1000 PY, 95% CI: 2.75-14.60) and 10.3-fold (35/1000 PY vs. 3/1000 PY, 95% CI: 1.18-123.07) higher among HIV-infected infants compared to HIV-exposed, uninfected infants, respectively (Table 2).

### Reasons for Hospitalization

Hospitalization was more often due to infectious morbidity than non-infectious morbidity (56% vs. 44%) (Table 3). Together - gastroenteritis, pneumonia and sepsis/meningitis accounted for 63% of infectious hospitalizations and 36% of total hospitalizations. Among hospitalized infants 5 had dual diagnoses of sepsis and pneumonia and 5 had sepsis and gastroenteritis. Abnormal laboratory values accounted for the majority of non-infectious hospitalizations, particularly abnormal bilirubin in the neonatal period.

**Table 3:** List of reasons for 381 hospitalizations among 737 HIV-exposed infants at 12 months of life in Pune, India

	N (%)		N (%)
Infectious	215 (56.0)	Non-infectious	166 (44.0)
Gastroenteritis	67 (17.6)	Hyperbilirubinemia	35 (9.2)
Meningitis or sepsis	44 (11.5)	Abnormal chemistry values†	32 (8.4)
Pneumonia	25 (6.6)	Neutropenia	21 (5.5)
Rash without fever	19 (5.0)	Anemia	12 (3.1)
Syphilis	18 (4.7)	Prematurity, low birth weight, small for gestational age	12 (3.1)
Conjunctivitis	15 (3.9)	Congenital anomaly	11 (2.9)
Impetigo	7 (1.8)	Respiratory distress syndrome	11 (2.9)
Upper respiratory infection	7 (1.8)	Meconium aspiration	8 (2.1)
Fever	5 (1.3)	Seizure	6 (1.6)
Tuberculosis	5 (1.3)	Failure to thrive	4 (1.0)
Cellulitis	3 (0.8)	Thrombocytosis	2 (0.5)
Hepatitis	1 (0.3)	Other skin conditions	2 (0.5)
Urinary tract infection	1 (0.3)	Trauma/abuse	2 (0.5)
Skin abscess	1 (0.3)	Irritability or colic	3 (0.8)
		Bleeding	1 (0.3)
		Umbilical granuloma	1 (0.3)

NOTES:

for infants with multiple diagnoses, infectious diseases were listed as the primary reason for hospitalization.

†Includes abnormal liver or renal function tests.

The 18 infants hospitalized for syphilis were admitted for treatment based on maternal syphilis serological diagnoses. The 15 infants hospitalized for conjunctivitis and 7 for upper respiratory infection were admitted for symptoms assessment, respectively; no other diagnoses were reported at discharge. Among hospitalized infants 5 had dual diagnoses of sepsis and pneumonia and 5 had sepsis and gastroenteritis.

### Risk Factors for Hospitalization

In adjusted analysis, infant HIV infection, advanced maternal age, and younger gestational age at birth predicted all-cause hospitalization, while female gender was protective (Table 4). There were too few pneumonia and sepsis/meningitis-related hospitalization events to adequately evaluate risk factors for these. Compared to >6 months of any breastfeeding, shorter breastfeeding duration of <4 or 4-6 months was associated with 2.44-fold (95% CI: 1.29-4.64) and 2.39-fold (95% CI: 0.94-6.10) increased risk of gastroenteritis-related hospitalization, respectively (adjusted for infant HIV status, cotrimoxazole use, and maternal AIDS)(data not shown).

**Table 4:** Risk factors for all-cause hospitalization using Poisson regression among 737 HIV-exposed infants at 12 months of life in Pune, India

	Univariate			Multivariate		
	IRR	95% CI	P-value	IRR	95% CI	P-value
**Infant Characteristics**						
HIV status						
HIV-infected	1.82	1.42-2.33	<0.001	1.52	1.14-2.02	<0.05
HIV-exposed, uninfected	1.00					
Female gender	0.73	0.60-0.90	<0.05	0.73	0.59-0.91	<0.05
Gestational age						
<37 weeks	1.93	1.48-2.52	<0.001	1.55	1.11-2.17	0.01
≥37 weeks	1.00					
APGAR at 5 minutes	1.01	1.001-1.008	0.02	1.00	1.00-1.01	0.16
Birth weight						
<2500 grams	1.35	1.09-1.67	<0.05	1.12	0.86-1.46	0.40
≥2500 grams	1.00					
Breastfeeding duration						
<4 months	1.04	0.83-1.29	0.75	1.06	0.85-1.34	0.60
4 - 6 months	0.98	0.69-1.41	0.93	1.01	0.70-1.47	0.94
>6 months	1.00					
Cotrimoxazole use						
WHO-guided	1.14	0.94-1.40	0.19	1.05	0.83-1.32	0.68
Non WHO-guided	1.00					
Extended-dose nevirapine						
Yes	0.99	0.81-1.20	0.89	0.97	0.79-1.19	0.75
No	1.00					
Infant HAART^†^						
Yes	2.20	1.37-3.53	<0.05			
No	1.00					
**Maternal Characteristics**						
Age (years)	1.06	1.03-1.09	<0.001	1.04	1.00-1.07	0.03
Gravidity	1.11	1.00-1.22	0.04	1.04	0.93-1.16	0.49
Marital status						
Not married	1.26	0.71-2.24	0.43			
Married	1.00					
Education						
Primary (<6 years)	0.80	0.65-0.98	0.04	1.32	0.87-1.99	0.19
Secondary (6-10 years)	0.72	0.49-1.07	0.11	1.13	0.76-1.70	0.54
Tertiary (>10 years)	1.00					
Religion						
Hindu	1.05	0.82-1.35	0.68			
Non-Hindu	1.00					
Housewife						
Yes	0.78	0.61-0.99	0.04	0.88	0.68-1.13	0.31
No	1.00					
Delivery type						
Vaginal	1.22	0.93-1.59	0.15			
Surgical	1.00					
Hemoglobin^¥^						
Severe (<7 g/dl)	2.66	1.74-4.05	<0.05	1.24	0.85-1.83	0.27
Moderate (7-<10 g/dl)	1.21	0.92-1.60	0.17	1.18	0.93-1.51	0.17
Mild (10-11 g/dl)	1.32	1.04-1.67	0.02	1.22	0.89-1.66	0.22
Normal (>11 g/dl)	1.00					
CD4 cell count^¥^						
<200 (cells/mm^3^)	1.93	1.40-2.67	<0.05	1.17	0.82-1.68	0.39
≥200 (cells/mm^3^)	1.00					
Viral load (Log 10)^¥^						
≥5 log (copies/ml)	1.38	1.09-1.74	<0.05	1.15	0.89-1.47	0.28
<5 log (copies/ml)	1.00					
Maternal HAART^†^						
Yes	1.55	1.15-2.09	<0.05	1.31	0.94-1.83	0.11
No	1.00					
Maternal AZT*						
Yes	0.86	0.69 - 1.07	0.17			
No	1.00					
Maternal NVP*						
Yes	0.66	0.54-0.81	<0.05	0.85	0.68-1.07	0.16
No	1.00					

NOTES:

^†^HAART use among HIV-infected mothers or infants at any time during the study. Infant HAART use was not included in the multivariate model as it was only applicable to HIV-infected infants.

^¥^Reported values are closed to delivery.

*AZT = zidovudine for PMTCT, NVP = nevirapine (intrapartum, single-dose) for PMTCT.

## Discussion

In this study, nearly 50% of HIV-infected Indian infants were hospitalized by 12 months of life at almost twice the hospitalization rate of HIV-exposed, uninfected Indian infants. Infections were the predominant reason for hospitalization, and gastroenteritis was the leading infectious cause of infant hospitalization in our cohort, which was counseled to exclusively breastfeed and abruptly wean at four months of life (revised to six months towards the end of the study). Sepsis/meningitis and pneumonia-related hospitalizations accounted for lower than expected proportion of hospitalizations, and gastrointestinal-related hospitalizations appeared to peak around 9 months of life. As expected, infant HIV status was a significant predictor of hospitalization risk along with prematurity and advanced maternal age. In-hospital mortality was 10-fold higher among HIV-infected infants compared to HIV-exposed, uninfected infants.

There are limited comparable hospitalization data among HIV-infected infants in other resource-limited settings. A South African study of HIV-infected children <5 years (67% <1 year) not on ART reported a hospitalization frequency of 47.9% [19], nearly identical to our finding of 47%. Another similar South African study of HIV-infected infants (birth-3 months) reported a lower hospitalization frequency of 18% and a rate of 2.33/1000 PY [20]. These comparisons are limited by age at outcome, breastfeeding details, and cotrimoxazole prophylaxis. Among HIV-exposed, uninfected infants not on ART, the following hospitalization frequencies have been reported: 5% by 3 months of life in South Africa [20], 6% at 12 months of life in Zambia [8], 17% at 6 months of life in Latin America [9], and 20.4% at 12 months among children <5 years (67% <1 year) in South Africa [19]. Overall, our hospitalization findings among HIV-infected infants appear similar to those of South Africa, which bears the highest worldwide burden of HIV infection. Some key differences in our cohort was that approximately half of them were on a short course antiretroviral prophylaxis regimen, majority were breastfed and all were participants in a clinical trial; however similar to the above-mentioned studies very few of the HIV-infected infants received HAART. Therefore some of these differences may make it difficult to make a complete comparison across studies.

As with all-cause hospitalization, there are few data available on cause-specific hospitalization. Furthermore, the available data, particularly hospitalizations due to infections, are limited by lack of pathogen specificity and comparisons across studies are hampered by different methods of ascertainment and duration of follow-up. Among categories of infection, we found a higher frequency of gastroenteritis-related hospitalization in our cohort compared to that reported in sub-Saharan African studies. A South African study of HIV-infected infants (birth-3 months) reported 7.7% of gastroenteritis-related hospitalization [20], compared to our 17% at 12 months. A Malawian study reported a 3.1% of gastroenteritis-related hospitalization [21] among HIV-exposed, uninfected infants (3-9 months of life), compared to our 7.6% at 12 months. One potential explanation for higher rates of gastroenteritis-related hospitalization in our cohort may be the common Indian practice of mixed feeding and animal milk substitutes for infant feeding, which we have previously observed [22,23]. A limitation of our study however is that the precise details of breastfeeding was not known at the time of this publication, but mixed feeding and milk substitutes also likely contributed to our high rates. Abrupt weaning is also likely to be an important contributor. Abrupt weaning in a large Zambian study was associated with significantly increased risk of HIV infection and gastrointestinal illness [24]. Furthermore, breastfeeding for shorter duration (<4 and <4-6 months) was associated with an over 2.0-fold increased risk of gastroenteritis-related hospitalization compared to >6 months. Similar results were found in Zambia where breastfeeding for <5 months among HIV-infected infants were associated with increased mortality compared to longer breastfeeding [24]. Because of such data [21,24] abrupt breastfeeding cessation is no longer recommended by the WHO and exclusive breastfeeding for at least 6 months is recommended to protect infants against excessive morbidity and mortality when infant formula is not affordable, feasible, acceptable, sustainable or safe [25]. The low rates of pneumonia-related hospitalization could be related to relatively high rates of cotrimoxazole use, even if not strictly according to WHO guidelines.

Our observed in-hospital mortality rates (with 16% of infants on ART) were lower than what has been observed in studies from sub-Saharan Africa (among infants not on ART). In South Africa, 13.2% [26] and 16.5% (children <5 years with 67% <1 year) [19] HIV-infected infants died in-hospital compared to our observed 6.8%. Similarly, higher proportions of in-hospital mortality have been reported among South African HIV-exposed, uninfected infants- 5.2% [26] and 4.6% (children <5 years with 67% <1 year) [19] compared to 0.9% seen in our study. The remaining 22 of 27 deaths occurred outside the hospital. Most of these occurred in the home, presumably with infants succumbing to illnesses before being able to be brought to the hospital for care.

The lower overall and in-hospital mortality seen in our study may be explained by the increased availability of health care services provided in a clinical trial setting, as none of the comparative studies were part of a clinical trial. In addition, many of our hospitalizations in the non-infection category occurred in response to abnormal laboratory values, some of which were likely triggered because of the increased monitoring that occurred within a clinical trial. Nonetheless, the frequency of in-hospital death among both HIV-infected and HIV-exposed, uninfected infant groups highlights the need for aggressive management of hospitalized infants and the importance of preventive measures.

Consistent with known predictors of infant morbidity, older maternal age, infant HIV infection, and prematurity predicted all-cause hospitalization. Interestingly, female gender was protective of all-cause hospitalization, which is consistent with reports of greater female infant immunity against infection and lower mortality from infections than among male infants in all income settings [27]. In contrast to other studies, we did not identify maternal CD4, viral load, or HAART use to be risk factors for infant hospitalization. This is possibly explained by differential health parameters, such as the low percentage of mothers with AIDS in India (7% in this study) compared to African studies (20-30%). Although extended nevirapine has previously been shown to reduce mortality in HIV-exposed breastfed infants [12], we found little data to suggest that it was mediated through a reduction in hospitalization or in-hospital mortality rates.

This study had several limitations. Since it analyzed a secondary endpoint of the parent study, the ability to detect statistical differences among secondary endpoints according to HIV status was limited. Little microbiological data were available to support most infectious diagnoses in this study, but WHO-guided probable and presumed diagnoses were used to standardize morbidity definitions [14]. Our data were generated in the context of a clinical trial setting and therefore may have underestimated the rate of infant hospitalization in the community as follow-up was frequent and many tertiary care services were readily available. Lastly, infant vaccination records (other than BCG given at birth) were not routinely collected in this study, and may have had an impact on frequency and distribution of hospitalization morbidity. However, despite these limitations, the data were carefully collected at several time points within the context of a clinical trial and provide among the best longitudinal data on hospitalization rates from HIV-exposed Indian infants.

## Consclusions

In conclusion, HIV-exposed Indian infants, especially HIV-infected Indian infants, experienced a high burden of hospitalization morbidity and mortality. Infections accounted for over half the hospitalizations with gastroenteritis being the most common cause among this HIV-exposed infant population. It is therefore critical to scale-up HIV PMTCT programs and enhance implementation of effective vaccinations, cotrimoxazole prophylaxis, prenatal care, and safe infant feeding practices which have all been shown to reduce infectious morbidity and all-cause mortality among HIV-exposed, infants.

## Competing interests

The author declares that they have no competing interests.

## Authors' contributions

HKS, RB and AG conceived of the study, participated in its design and coordination, and helped draft the manuscript. NG and UM performed the statistical analysis and data management. AK, RB, JS, NS, ST, RP, and AJ participated in acquisition of data and revision of the manuscript. All authors have given final approval of the version to be published.

## Pre-publication history

The pre-publication history for this paper can be accessed here:

http://www.biomedcentral.com/1471-2334/11/193/prepub
